# Editorial: Expanding horizons in stress research—innovative paradigms and new directions in 2025

**DOI:** 10.1016/j.ynstr.2026.100803

**Published:** 2026-03-20

**Authors:** Mathias V. Schmidt, Lawrence Reagan

**Affiliations:** a Research Group Neurobiology of Stress Resilience, Max Planck Institute of Psychiatry, Munich, Germany; b University of South Carolina School of Medicine, Department of Pharmacology, Physiology, and Neuroscience, Columbia, USA; c Columbia VA Health Care System, Columbia, USA

## 1 Introduction

The research featured in *Neurobiology of Stress* throughout 2025 highlights a period of remarkable expansion, unveiling exciting new avenues that deepen our understanding of the resilient and vulnerable brain. As we navigate an era defined by rising stress and challenges, the work highlighted in this editorial illustrates that stress is not a monolithic experience, but a dynamic interplay between genetic vulnerability and environmental timing. By synthesizing findings across four core themes—ranging from the lasting programming of early-life adversity to the complex neurobiology of social dominance—we present a landscape of research that not only elucidates the mechanisms of vulnerability but also identifies novel avenues for fostering resilience in an increasingly stressed world.

## 2 Theme 1: The developmental blueprint: early-life adversity and programming

A main theme that emerged is how stress during critical prenatal or postnatal windows permanently alters neural architecture and behavior. For example, evidence suggests that prenatal stress causes transient but impactful shifts in GABAergic cell populations that lead to permanent behavioral abnormalities (Shoshani-Haye et al., 2025). Similarly, adolescent amygdala activity interacts with early-life stress to fundamentally rewire corticolimbic connectivity (Cody et al., 2025). Furthermore, Chelini et al. reveal that early-life adversity exacerbates behavioral deficits in mice with a *CNTNAP2* genetic predisposition (Chelini et al., 2025). Finally, research into paternal combat trauma provides evidence for intergenerational transmission, affecting the behavioral and brain functions of the next generation (Yamakawa et al., 2025). Collectively, these studies represent a landmark shift in stress neurobiology by illustrating how stress acts as a biological architect across the lifespan—and even across generations—utilizing specific molecular pathways to recalibrate neural circuitry. By integrating genetic vulnerabilities with environmental stressors during critical developmental windows, this research moves the field toward a unified understanding of how early-life “sculpting" of the brain creates a lasting template for psychological and physiological vulnerability. These programming events have long-lasting impacts which are very prominent during aging, and may even be used to predict brain age (Fleming et al., 2025; Jeong et al., 2025).

## 3 Theme 2: The social brain: Isolation, aggression, and dominance

Another compelling area addressed by a number of publications in Neurobiology of Stress last year reflects the profound impact of social environment and peer interaction on the adult stress response. Findings indicate that social isolation specifically alters opioid receptor expression in the locus coeruleus to drive affective behavioral changes (Tkaczynski et al., 2025). Within the infralimbic cortex, distinct neural ensembles appear to dictate whether an individual adopts a vulnerable or resilient coping strategy during social defeat (Laymon et al., 2025). Szebik et al. reveal that aggressive social encounters evoke rapid, dynamic shifts in serotonin transporter expression in the prefrontal cortex (Szebik et al., 2025), a finding complemented by John et al., who show that the mere threat of aggression is a primary trigger for activating orexin-modulated neurocircuits that determine stress vulnerability (John et al., 2025). Scott et al. expand the scope of these findings by documenting the severe cardiometabolic and autonomic consequences—including sustained hypertension—that stem from chronic social defeat (Scott et al., 2025). Collectively, these papers provide a comprehensive view of social stress as a multi-systemic force that reorganizes both the brain and the body, moving the field toward a deeper understanding how stressful experiences create a lifelong template for both psychiatric and physiological health.

## 4 Theme 3: Beyond the brain: The gut-microbiota-brain and vascular axis

Expanding the scope of stress research beyond the central nervous system, recent studies have increasingly focused on the systemic nature of stress as a dialogue between the brain, the gut-vascular barrier, and metabolic states. For example, Wang et al. identified a novel direct neural circuit from the insular cortex to the nucleus tractus solitarius that drives acute stress-induced gastric mucosal damage (Wang et al., 2025), while Aghighi Bidgoli et al. show that probiotics can rescue cognitive deficits by simultaneously restoring tight junction integrity in both the hippocampus and the intestines (Bidgoli et al., 2025). Furthermore, one study revealed that high-intensity interval training (HIIT) mitigates cognitive decline in chronically stressed mice by targeting the blood-brain barrier, specifically alleviating the repression of the seal-protein *Cldn5* caused by elevated homocysteine (Sun et al., 2025). Finally, Kördel et al. provide a comprehensive meta-analysis showing that an individual's baseline metabolic state—including glucose levels and adiposity—fundamentally shapes their hormonal cortisol reactivity to acute challenges (Kördel et al., 2025). Collectively, these findings underscore that the neurobiology of stress is inseparable from the body's physical “borders," where the permeability of the gut and blood-brain barriers serves as a critical interface for pathology. This body of work marks a pivotal direction for the field, suggesting that metabolic and peripheral health are not merely secondary effects of stress, but are central components of the systemic feedback loops that determine resilience and vulnerability.

## 5 Theme 4: Individual variability: Sex differences and resilience

Building on the systemic complexity of the stress response, current research has increasingly sought to decode why individuals diverge into resilient or vulnerable phenotypes, with a specific focus on sexual dimorphism and region-specific molecular markers, a theme that also resonated a lot in many of the papers published last year in Neurobiology of Stress. In females, microglial MyD88 signaling in the prefrontal cortex acts as a sex-specific mediator of anxiety, where its ablation rescues behavioral dysfunction (Rawls et al., 2025). Further sexual dimorphism is evident in PACAP signaling in the infralimbic cortex, which differentially modulates social and threat behaviors in male and female rats (Smail et al., 2025), a finding echoed by Fjerdingstad et al., who link anxiety susceptibility to sex-specific timing in the maturation of perineuronal nets (PNNs) within the reward circuits of the habenula (Fjerdingstad et al., 2025). On the level of biological signatures, Long et al. use transcriptomic profiling to reveal that resilience is not a passive state but an active molecular process in the hippocampus and amygdala, characterized by distinct insulin and plasticity-related gene signatures (Long et al., 2025). These findings are complemented by Zonca et al., who uncover shared biological signatures of vulnerability across the habenula and insular cortex that are uncovered by early-life stress in both sexes, and Brivio et al., who find that stable autophagy and mitophagy dynamics in the ventral hippocampus are essential for maintaining a resilient phenotype during chronic mild stress (Brivio et al., 2025; Zonca et al., 2025). Collectively, these papers highlight that resilience is an active process, that is highly sex-dependent and illustrates that personalized interventions aimed to increase resilience will need to be different in women and men.

## 6 Outlook

The research published in 2025 in Neurobiology of Stress reflects a steady progression toward a more integrated understanding of how stress affects the whole organism. By moving beyond purely brain-centric models to include the brain-body interactions, by further defining stress circuitry and long-term signatures, and by better understanding variables that impact individual resilience like biological sex, the field is gaining a clearer picture of the diversity behind stress responses and their consequences for health and disease. These studies suggest that future interventions will need to consider an individual's genetic background, developmental history and physiological profile, potentially allowing for more targeted approaches to building resilience. As editors of Neurobiology of Stress, we are encouraged by the steady mechanistic and translational insights provided by the contributions to our journal this past year. We look forward to seeing how these themes evolve and are eager to continue supporting the community as it works to untangle the complexities of stress and recovery.

## Declaration of competing interest

Given their role as editor in chief of Neurobiology of Stress, Mathias V. Schmidt and Lawrence Reagan had no involvement in the peer review of this article and had no access to information regarding its peer review. Full responsibility for the editorial process for this article was delegated to another journal editor.

## CRediT authorship contribution statement

**Mathias V. Schmidt:** Writing – original draft. **Lawrence Reagan:** Writing – review & editing.

## Data Availability

No data was used for the research described in the article.
